# Virtual reality-based music attention training for acquired brain injury: A protocol for randomized cross-over trial

**DOI:** 10.3389/fneur.2023.1192181

**Published:** 2023-08-08

**Authors:** Joon-Ho Shin, Eunju Jeong

**Affiliations:** ^1^Department of Rehabilitation, National Rehabilitation Center, Ewha Womans University, Seoul, Republic of Korea; ^2^Department of Music Therapy, Graduate School, Ewha Womans University, Seoul, Republic of Korea

**Keywords:** music therapy, attention deficit, acquired brain injury, virtual reality, attention evaluation and training, neurocognitive rehabilitation

## Abstract

Attention training is the primary step in the rehabilitation for patients with acquired brain injury (ABI). While active music performance has been reported to aid neural and functional recovery, its efficacy for patients with ABI remains uncertain due to methodological concerns. The purpose of the study is to develop a virtual reality-based music attention training (VR-MAT), which utilizes a visually guided, bilateral drumming in an immersive environment to train attention and executive functions. We also aims to examine the feasibility and effectiveness of the VR-MAT with a small sample size of participants (3–60 months after ABI, *N* = 20 approximately). Participants will be randomly assigned to either a waitlist control or music group, in which VR-MAT will take place five times weekly over 4 weeks (randomized crossover design). The evaluation of VR-MAT performance will include accuracy and response time in music responses. Neurocognitive outcome measures will be administered to quantify pre-post changes in attention, working memory, and executive functions. Additionally, functional near-infrared spectroscopy will be employed to explore the relationships between musical behavior, neurocognitive function, and neurophysiological responses.

## 1. Introduction

Acquired brain injury (ABI) is the damage to the brain that occurs due to certain events after birth (1) and includes traumatic injury and/or stroke (2, 3). Many individuals with ABI experience temporary or permanent neurological sequelae which is not limited to psychomotor, cognitive, and communication difficulties (4–7). The prevalence of ABI-related changes in cognition, including attention, working memory, memory, and executive function, is high (8–12), leading to a lower quality of life, accelerated functional decline, and a higher risk of dependence and mortality (5, 7). Cognitive impairments can decrease therapeutic potential and clinical benefits, since verbal and non-verbal information given in therapeutic settings is hardly recognized. Attention impairment should be a priority in the rehabilitation agenda for patients with ABI because dysfunctions in the fundamental mechanism of attention are pervasively involved in cognitive impairment (13–19). More importantly, auditory attention appears to be more challenging in patients with ABI (20, 21). These patients often have difficulty attuning their attention to relevant auditory information and distinguishing target sounds from distracting sounds (20, 22–24).

Auditory information processing is challenging because it is both sequential and simultaneous. Listening to music can enhance attentional function, which is required to process complex auditory information. Music automatically activates the attention system, even when actively ignoring presented melodies and voluntarily attending to target melodies (25). In addition, music processing in different textures can lead to activation in neural regions that house different types of attention, such as selective attention and divided attention, depending on the manner of listening to music (i.e., holistic, selective, or divided) (26–28). Music error/change detection tasks can be utilized to investigate and train auditory attention (26, 29–33). More recently, active music performance and listening have been applied to cognitive rehabilitation. An initial study investigated the effects of a music attention training program on five adults with TBI (34), which was developed based on a hierarchy of attention (35). Furthermore, one study examined the effect of a music attention training program on alternating attention using a dichotic listening test (36), and another study suggested that music therapy is an effective early intervention for cognitive rehabilitation (37).

More recently, active music performances have been applied to various populations, such as juveniles, adolescents with neurodevelopmental disorders, and psychiatric patients. Abrahams and van Dooren (38) compared music attention control training (MACT) with non-standardized music therapy (NSMT) and treatment as usual (TAU) in terms of the attentional ability of juveniles (*N* = 6). With a six-week training of 45 min weekly, some participants in the MACT group showed an extensive increase in selective, sustained, and divided attention. Pasiali et al. (39) investigated the effect of the MACT on the attentional function of adolescents with neurodevelopmental delays using a single-group pre-and post-test design. With a six-week training of 45 min weekly, participants showed considerable improvements in selective attention and divided attention. van Alphen et al. (40) conducted a randomized controlled trial to determine the effect of MACT on attention skills in a group of adult psychiatric patients. After a 6 week training of 30 min weekly, single-blind pre-and post-intervention showed a marked enhancement in selective, sustained, and alternating attention in the MACT group. Jones (14) conducted a randomized controlled trial (RCT) to examine the effect of MACT compared to that of Attention Process Training (APT). The findings showed significant values and partial support from nonparametric testing, suggesting the potential of MACT in favor of APT.

Despite rigorous attempts, the efficacy of active music performance for patients with ABI is still under debate. For instance, in a systematic review of music and rehabilitation, Bradt et al. (41) reported that psychomotor rehabilitation effectively improves gait regulation, yet the success of cognitive rehabilitation remains uncertain. Similarly, Magee et al. (42) provided evidence supporting the positive influence of music interventions on upper and lower mobility functions as well as speech. Nonetheless, only two studies (43, 44) demonstrated the efficacy in music-based cognitive rehabilitation. Both reviews agreed on the urgent need for high-quality RCTs focusing on cognitive rehabilitation, along with a lack of replicable therapeutic protocols for music-based training designed to aid recovery from cognitive deficits (34, 36, 45). Most studies relied solely on functional outcome measures without quantifying musical responses, potentially missing key relationships between musical and functional behaviors, as well as changes uniquely evident through musical behaviors. To address the limitations, we developed a replicable therapeutic protocol of active music performance and integrated the protocol with a virtual reality system. The efficacy of VR-MAT will be evaluated through randomized cross-over trials, using a comprehensive data collection strategy that incorporates neuropsychological, neurophysiological and music responses measures.

## 2. Materials and methods

### 2.1. Study design

The present study will be conducted as an assessor-blinded, randomized, controlled trial with a crossover design at a rehabilitation hospital. Participants will be randomly allocated to either the treatment sequence: VR-MAT first and second with conventional cognitive training (CT) (VC group), or in the opposite order (CV group) with an allocation ratio of 1:1 using a computer-generated randomization table. The crossover design is selected because the participants need to serve as their own control group to minimize confounding effects on cognition, which might be markedly affected by a person’s traits, which is not intended by the intervention. In addition, it might be beneficial for the statistical efficiency of this pilot trial, in which VR-MAT had not been attempted for patients with ABI.

Each VR-MAT or CT training period will last 4 weeks with five 30-min intervention sessions per week, resulting in a total of 20 sessions. Therefore, each participant will have received a total of 40 sessions of the intervention, comprising 20 sessions of VR-MAT and 20 sessions of CT. The randomized group assignments were concealed in consecutively numbered sealed opaque envelopes placed in a plastic container. The allocated group will then be determined by the sequential opening of the envelopes after obtaining written informed consent from the participants. Participants will receive a conventional inpatient rehabilitation program (1 h of physical therapy or 30 min of occupational therapy); however, they will not receive a cognitive intervention. Outcome measurements will be performed by a researcher blinded to the group assignment.

### 2.2. Participants

Participants will be recruited among patients who have been admitted to the neurorehabilitation unit of the rehabilitation hospital. The inclusion criteria are as follows: (1) cognitive impairment secondary to a first-ever ABI, as evidenced by a Clinical Dementia Scale (CDR) score of 0.5 – 1; (2) a diagnosis of ABI > 3 months ago with medical records and/or brain imaging; (3) no history of prior ABI; (4) upper limb muscle strength of ≥ 3 on the medical research council scale, indicating suitability for training and assessment; (5) age >19 years. The exclusion criteria are: (1) known cognitive impairment prior to ABI; (2) severe aphasia or cognitive communication disorder precluding cognitive intervention, including MAT and CCT, or assessment; (3) neurological or psychiatric disorders other than ABI; (4) severe depression (Geriatric Depression Scale-short form score of ≥8); (5) uncontrolled medical illnesses, such as severe cardiac or respiratory disease. The exclusion criteria are as follows: (1) known cognitive impairment prior to ABI; (2) severe aphasia or cognitive communication disorder which precluded cognitive intervention including MAT and CCT or assessment; (3) neurological or psychiatric disorder other than ABI; (4) severe depression [Geriatric Depression Scale-short form ≥8]; (5) uncontrolled medical illness such as severe cardiac or respiratory disease. The experimental protocol was approved by the ethics committee of the rehabilitation hospital (IRB no. NRC-2015-04-032). Sample size calculation was not possible because the effects of the VR-MAT on cognitive function have not yet been reported. Therefore, we have set the sample size to 12 for each arm, in accordance with the minimum number recommended for pilot trials (46, 47). In total, considering a potential participant attrition rate of 20%, we aim to recruit 24 participants for this pilot study.

### 2.3. Virtual reality-based music attention training

**Figure 1:**
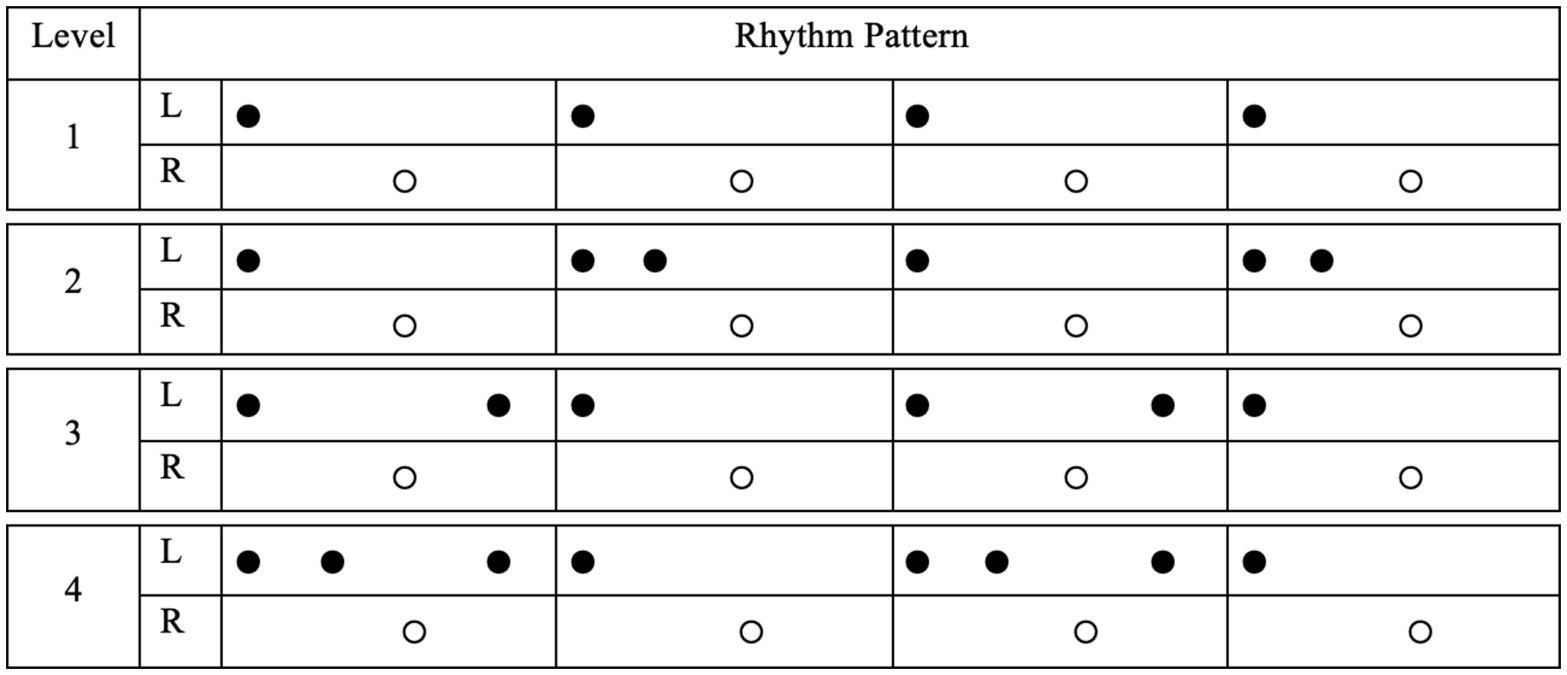
Examples of rhythm pattern across difficulty level.

VR-MAT comprises virtual reality-based, visually guided, bilateral drumming on electronic instruments. The VR-MAT is designed to fit individual therapeutic sessions and employs the patients’ preferred songs given in a multisensory environment (i.e., visual, auditory, and haptic). The VR-MAT consists of evaluation and training sessions. The VR-MAT-Evaluation includes baseline tempo and visuomotor response assessment tasks, aiming to ascertain individual variations, providing baseline information for VR-MAT-Training. In the baseline tempo assessment, participants will be asked to play the drums at a comfortable tempo for 10 s using their dominant and non-dominant hands independently. The baseline tempo assessment will be repeated thrice. Once the baseline tempo of the patients’ drumming is calculated, the participants will be asked to play a visually cued rhythmic pattern on the given electronic drum. The rhythm patterns will consist of four-beat rhythmic patterns with a binary meter, representative of four popular music genres, Rock, Swing, Shuffle, Bossa-Nova. Each genre will encompass rhythmic patterns that vary in the level of difficulty. Figure 1 presents examples of rhythm pattern varied across difficulty level within the Rock genre. Visual cues will be presented at a comfortable tempo via flashes on a computer screen, positioned 70–80 cm in front of the patients. In the VR-MAT-Evaluation, we will also collect information about participants’ previous musical experiences, including years of music training, types of musical activities they have participated in, and their musical preferences.

VR-MAT-Training consists of four levels of visually guided drumming tasks designed to restore various attention types in a hierarchical manner: (1) MAT-Level 1: One hand drumming, (2) MAT-Level 2: Two hand drumming, (3) MAT-Level 3: Reversed drumming, and (4) MAT-Level 4: Alternating drumming. In VR-MAT Training Level 1, a four-beat rhythm pattern is visually presented on the computer screen, first on the left side, and then independently on the right side. In VR-MAT Training Level 2, the visual cues used in Session Level 1 are combined and presented concurrently. VR-MAT-Training Level 3 is a two-hand drumming task that employs reversed rhythm patterns compared with those used in Level 2. For example, the visual patterns presented on the right side are presented on the left side and vice versa. Because individuals already possess habituated body patterns, they must inhibit previously learned patterns while learning new reverse patterns. Finally, VR MAT Training Level 4 provides the rhythm patterns of Levels 2 and 3 in a randomly alternating manner. Each task includes 20–21 trials, and the completion time is approximately 40 min.

**Figure 2:**
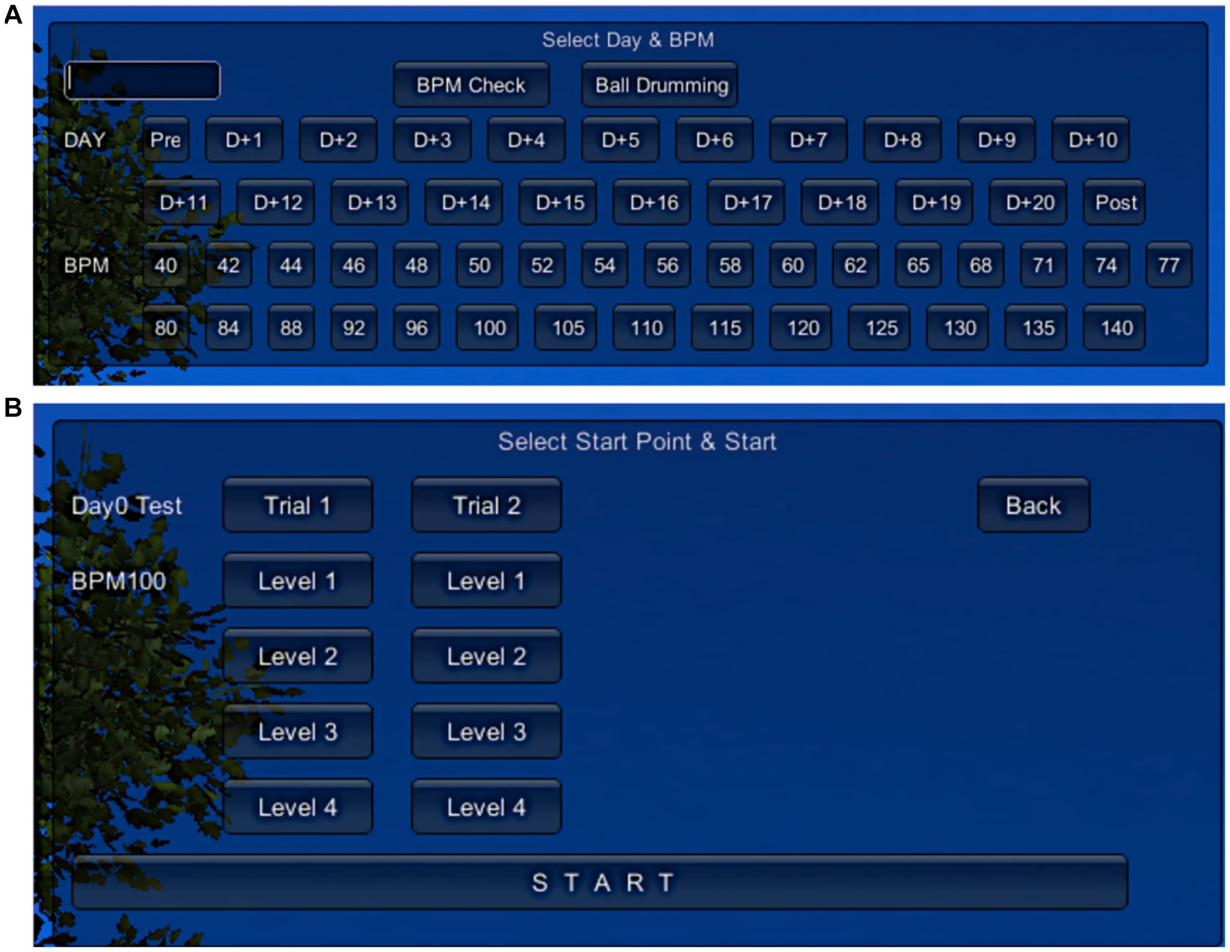
User interface of VR-MAT. **(A)** The first page of the UI includes four sections; User ID input, Warm-up exercise (BPM check, Free ball drumming), Day, and BPM selection; **(B)** The second page of the UI includes selection of trials and levels.

**Figure 3:**
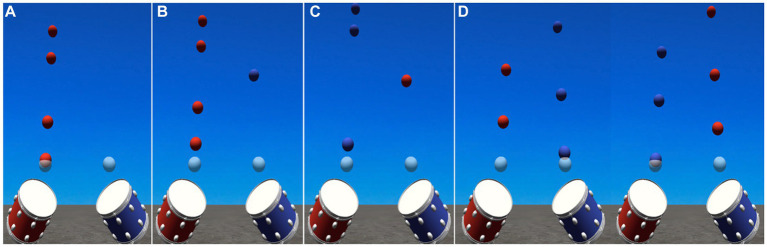
Examples of each of the four levels of training. **(A)** VR-MAT Level 1; One-hand drumming; **(B)** VR-MAT Level 2; Two-hand drumming, **(C)** VR-MAT Level 3; Two-hand drumming-reversed, **(D)** VR-MAT Level 4; Two-hand drumming-randomized.

Given the nature of bilateral drumming, the VR-MAT utilizes a familiar four-beat rhythmic pattern with a binary meter. Rhythmic patterns are carefully designed to vary the level of difficulty based on the VR-MAT-Evaluation results. Background music (BGM), originally composed of a binary meter, is selected from the patient’s preferred song list. The BGM can be played by a therapist or recorded without a vocal track. In both cases, adjustment of the BGM tempo is necessary to maintain the baseline tempo of the patients. With the auditory feedback (either a kicking or snaring drum sound), three types of visual cues are given: (a) if the drum stroke is falling within a time range of ±150 millisecond, a flashlight is given (i.e., matched), (b) if the drum stroke is hit earlier than the exact target point, ranging between −300 to −150 millisecond, the ball is exploded (i.e., mismatched), (c) if the ball is missed in the time range mentioned above, no visual cue is presented (i.e., unmatched). Figure 2 shows the user interface of VR-MAT, and Figure 3 presents example rhythm patterns for each of the four training levels.

#### 2.3.1. VR-MAT behavioral data

We will evaluate the VR-MAT performance four times [pre (day 1), mid1 (day 8), mid2 (day 15), and post (day 22) sessions]. We will obtain (1) the VR-MAT performance accuracy, which is defined as the number of matched strokes divided by the total number of strokes, and (2) the VR-MAT response time (RT), which is the time required for the matched strokes. No background music will be played during the evaluation sessions.

#### 2.3.2. Equipment set-up

**Figure 4:**
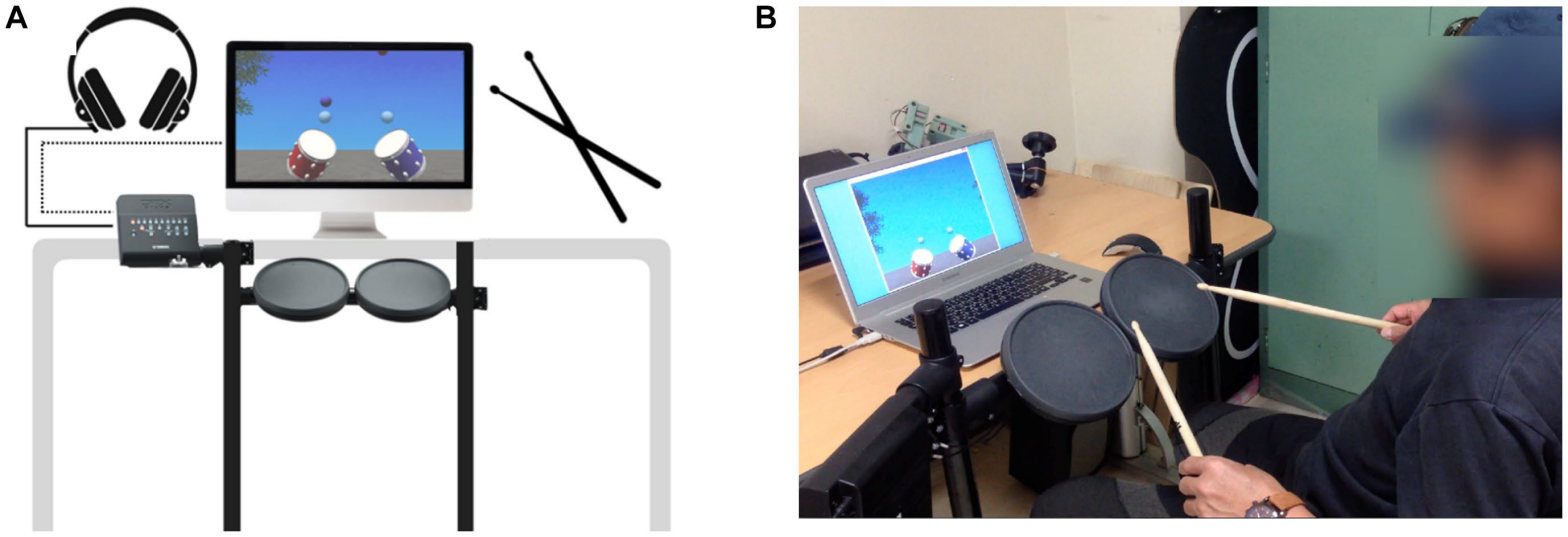
**(A)** Example of equipment set-up. A control panel of the electronic drum is connected to a desktop computer and a monitor. Accompaniment and sound feedback will be provided via a headphone that is connected to the control panel. **(B)** An individual demonstrating VR-MAT.

All musical stimuli will be generated using Logic Pro X (Apple Inc., California, United States) and combined with the visual stimuli using Unity (Unity Technologies, San Francisco, United States). The electronic drum consists of tom-toms on an adjustable stand, which is connected to a screen, speakers, and a desktop computer. A total minimum area of two meters squared will be required to set up the tom-toms on the stand, including space for the participant to sit (Figure 4).

### 2.4. Conventional cognitive training

The CCT was designed to mimic attention process training, which has proven to be effective in patients with ABI(48, 49). Therefore, the CCT encompasses various levels of attention, such as focused, sustained, selective, alternating, and divided attention, and progresses with the participants’ levels. The training difficulty level is adjusted by modifying tasks, such as altering the number of presented stimuli. Feedback is provided after each task, and the therapist provides a summary at the end of each session. The CCT is administered by an experienced occupational therapist, utilizing diverse training methods including cards, dictation, buzzer, paper-and-pencil tasks, or computerized cognitive training.

### 2.5. Outcome measurement

Baseline demographic characteristics, including sex, age, diagnosis of ABI, duration after ABI, side of brain lesion, and education, will be collected. Additionally, neuropsychological outcomes will be measured for each participant at baseline, after 20 sessions (4 weeks), and after 40 sessions of intervention (after 20 sessions of alternative intervention; 8 weeks) by a blinded neuropsychologist. We will collect data on neuropsychological outcomes in two ways: global cognitive function and executive function, which are specific targets of VR-MAT. All of the cognitive function tests will be administered in Korean. Additionally, we will obtain VR-MAT behavioral data, which is previously explained, and functional infrared spectroscopy (fNIRS) data (Table 1).

**Table 1:** List of outcome measurements.

Global neuropsychological assessment	Mini-mental status examination (MMSE)
Clinical dementia rating (CDR)
Global deterioration scale (GDS)
Subjective memory complaint questionnaires (SMCQ)
Executive function assessment	Trail making test (TMT)
	TMT-black & white (TMT-B&W)
	Attention-switching Task (AST)
	Stop signal task (SST)
	Spatial working memory (SWM)
VR-MAT behavioral measures	VR-MAT response time
	VR-MAT accuracy
Functional near-infrared spectroscopy	Oxygenated hemoglobin
	Deoxygenated hemoglobin
	Total hemoglobin concentration
	Oxygen level change

#### 2.5.1. Global neuropsychological assessment

We will administer the mini-mental status examination (MMSE) (48), clinical dementia rating (CDR) (49), and global deterioration scale (GDS) (50) for the global cognitive functions and subjective memory complaint questionnaires (SMCQ) (51) for subjective cognitive function by an experienced research neuropsychologist. The MMSE is the most widely used screening tool for cognitive deficits. It comprises 30 items that provide information about orientation for time and place, memory (encoding and delayed recall), attention, calculation, language, and visuospatial construction. The CDR is a semi-structured informant-based clinical interview that rates global cognitive performance and functioning. It comprises six domains (memory, orientation, judgment and problem-solving, community affairs, home and hobbies, and personal care) (49). The global CDR score (range 0–3) will be calculated using an established algorithm indicating dementia severity: 0, no dementia; 0.5, questionable dementia; 1, mild dementia; 2, moderate dementia; and 3, severe dementia. The CDR sum of the box (CDR-SOB; range, 0–18) will be obtained as the sum of each domain score. A higher global CDR or CDR-SOB score indicates a greater severity of dementia. The Korean version of the CDR has also been validated (52). The GDS, which emphasizes memory function and the ability to complete activities of daily living, is used to rate the severity of cognitive deterioration in seven stages ranging from “no global impairment” (stage 1) to “very severe global impairment” (stage 7). Stages 1–3 indicate pre-dementia and stages 4–7 represent dementia (8). In addition, we will measure subjective cognitive function (beliefs about one’s own memory) using a 14-item of SMCQ (subjective memory complaint questionnaire). The total SMCQ score ranges from 0 to 14, with higher scores indicating more severe subjective cognitive decline (51).

#### 2.5.2. Executive function assessment

Targeting executive function specifically, we will perform Trail Making Test (TMT) (53), TMT-Black & White (TMT-B&W) (54) as paper-and-pencil tests, and computerized cognitive function tests (Attention-Switching Task [AST], Stop Signal Task [SST], Spatial Working Memory [SWM]) from the Cambridge Neuropsychological Test Automated Battery (CANTAB) (55). Participants will be seated comfortably in front of a desk and will be asked to perform the tests. The TMT consists of parts A (TMT A) and B (TMT B), in which TMT A is related to visual attention skills and TMT-B is related to executive functions. The TMT-A requires participants to draw lines to connect randomly distributed numbers in ascending order, whereas the TMT-B requires participants to connect numbers and letters alternatively (56, 57). The TMT-B&W is similarly constructed. The TMT-B&W A will prompt the participants to connect the circles containing numbers in ascending order. In contrast, TMT-B&W B will prompt the participants to connect the numbers in consecutive order, alternating between the two color sets (black and white) (54). TMT is measured as the time taken to complete the task in parts A (TMT A) and B (TMT B), and we obtained TMT A, TMT B, TMT-B&W A, and TMT-B&W B from both tests. In addition, we obtained TMT B-TMT A by subtracting TMT A from TMT B, which reflects greater executive function, sequestering psychomotor speed, and visual search (58, 59).

The researcher will administer the CANTAB tests using a two-button press pad connected to a touchscreen tablet computer, following the administration guide. The AST reflects selective attention and cognitive flexibility, along with top-down executive function, measuring cued attentional set-shifting, which manages conflicting information and inhibits task-irrelevant information. In each trial, the participants will see instructions presented on the monitor about which stimulus they should respond to, either the placement (right or left on the screen) or the direction (to the right or left) of an arrow. The participants will then be asked to press the right or left button according to the presented arrow. Thus, some trials will present congruent stimuli (placement and direction are identical), whereas others will display incongruent stimuli (the side on which the arrow appeared), thereby assessing the ability to switch attention between the placement and direction of an arrow. There are two different conditions between the two consecutive trials: non-switched trials (previous and present trials are identical) and switched trials (previous and present trials are not identical). AST outcome measures include response time (RT) and the number of correct trials; latency in congruent, incongruent, and all conditions; and percent correct (rate of correct response). A lower RT or a higher number of correct trials indicate better performance. In addition, we will obtain the following variables: congruency cost (ms, RT in incongruent condition – RT in congruent condition) and switch cost (ms, RT in switched trial – Rt in non-switched trial), in which a lower cost indicates better executive function.

SWM reflects spatial working memory or executive function through a search task, which is the ability to retain and manipulate visuospatial information. In each trial, the participants will be presented with randomly located colored boxes on the computer screen and will be asked to search through those boxes by touching each box to reveal its contents and find all the hidden tokens, in which the number of hidden tokens corresponded to the number of boxes. When they find a yellow token inside the box, it will be moved to, and fill the column on, the right side of the screen. The yellow token is never hidden in the same box twice; thus, the participants will be told to try to find the next hidden token, open the unselected box, and not revisit the previously opened box until they find the token. It is considered an error if the participant opens any box that is already opened. When the participants find a token in each box, the trial ends. As the participants complete each trial, the number of boxes increases from four to six to eight boxes. To reduce errors, the participants must remember where the tokens were previously hidden. The outcomes of SWM are “between errors” and “strategy.” Between errors is defined as the number of revisits to the box in which the participants already found a yellow token in the previous search, with a lower value indicating higher performance. The strategy is the effectiveness of the participants’ searching, in which participants follow a pre-specified sequence beginning with a specific box and return to the same box to start a new sequence. It ranges from 8 to 56, with a higher score indicating noncompliance with a specific strategy and a poor strategy.

The SST measures an individual’s ability to inhibit a response that has already been initiated, in two stages (go and stop trials). (1) Go trial: The subjects are requested to make a speeded response by pressing a button depending on the direction of an arrow pointing (left button for a left-pointing arrow and right button for a right-pointing arrow). (2) Stop trial: If an audio tone (a beep signal) is presented, participants should withhold any response to the stimulus and not press the button. The SST outcome measures include stopped signal reaction time (SSRT) and the proportion of successful stops, which are calculated from the last half of the trials. The SSRT is an indirect estimate of the inhibitory response speed, indicating the time required to abort a pre-potent action in the presence of a stop signal (stop signal delay), which is calculated by subtracting the RT on the stop trial from the mean RT on the go trial. A lower SSRT indicates a faster response and better performance. The proportion of successful stops is obtained with the number of times of successful stops, in which the participant stopped successfully, divided by the total number of stop signals, with higher indicating better performance.

### 2.6. Functional near-infrared spectroscopy

We will also examine the hemodynamic changes using fNIRS. The hemodynamic responses will be obtained at a sampling rate of 0.65 s, and the data will be converted to concentrated changes of hemoglobin using the modified Beer–Lambert law. Subsequently, a zero-phase low-and high-pass filter with a cut-off frequency of 0.01–0.09 Hz will be applied using a matrix laboratory (MATLAB) (60–62). Additionally, we will employ vector-based phase analysis to examine multifaceted aspects of cerebral hemodynamic changes (63–66). Vector-based phase analysis is based on an orthogonal vector coordinate plane defined by four indices: oxygenated hemoglobin, deoxygenated hemoglobin, total hemoglobin concentration, and oxygen level changes in the blood vessels. The four indices will be subjected to the calculation of angle k, which indicates the degree of oxygen exchange (66–68), and a scalar L, which indicates the amount of hemoglobin variation (69), and will be further used to calculate the phase-associated response intensity (PRI).

### 2.7. Statistical analysis

We will perform descriptive statistical analysis of the general demographics of the study participants. We define completers as participants who finish 20 sessions of either intervention (VR-MAT or CT) and undergo measurements at 4 weeks of intervention. The statistical analysis will be conducted on an intention to treat basis and the last observation carried forward method will be used to complete the data. Adherence will be reported as the number of completers among enrolled participants. For neuropsychological assessments, a comparative analysis between two different intervention sequences (VC or CV) will be performed and the carry-over effects will be tested using *t*-tests on the change in each sequence for all crossover endpoints (70, 71). For variables with insignificant carry-over effects, the changes in variables during specific interventions from either sequence will be combined and compared. For patients with significant carry-over effects, we will compare the changes in specific interventions during the first period (20 sessions of either intervention). Comparisons between interventions will be performed using a two-tailed *t*-test or the Mann–Whitney *U* test, depending on normality. For music behavior responses obtained from the VR-MAT data, we will perform a two-way repeated-measures analysis of variance (ANOVA) on level (levels 1, 2, 3, and 4) and time (pre, day 8, day 15, and post) with Bonferroni *post-hoc* comparisons.

Repeated measures of correlation between executive function assessment and VR-MAT variables will be examined to determine the linear relationship between paired repeated data using the R package rmcorr from R.3.6.1 (72). The independent variables will remain the same for the analysis of fNIRS data, and the dependent variables will be the PRIs obtained from all channels. All analyses will be performed using the software package R.3.6.1,^1^ and the level of significance will be set to 0.05.

## 3. Discussion

Instrumental music playing combined with VR technology might be a promising strategy for increasing the effectiveness of MAT by providing individualized and systematic interventions. This study aims to develop a replicable therapeutic protocol using an active music-playing platform. This topic is essential, considering that individuals need customized training programs suited to their functional levels and musical preferences. In addition, an increasing number of individuals require remote access to music-based attention programs, specifically those with restricted mobility. Moreover, our system can be used as a cognitive function measurement tool because music behavior data will be recorded with regard to accuracy and response time across all sessions. The data will be used to set up an individualized rhythm, determine whether to proceed to the next level of trial, and track changes over time.

The effectiveness of the system will be explored using cognitive outcome measures, including global and specific cognitive function test, especially executive function. Attention is a key component of executive function, as attention direct cognitive resources toward more important stimuli in order to sustain information processing and engaging and disengaging attention is linked to flexibility or inhibitory control [3; 4]. Thus, our cognitive outcomes could demonstrate how improvements in attentional abilities impact executive function, moreover global cognitive function. In addition, fNIRS data employ multifaceted variables of cerebral hemodynamics to indicate a PRI, which can provide interpretable information in terms of relationships and intensity changes over time. Therefore, the present study will contribute to increasing the scientific rigor of understanding how active music playing can affect cognitive function and cerebral hemodynamic responses, thus demonstrating its efficacy in cognitive rehabilitation. This specific study protocol enables music therapists to measure and quantify musical responses and clearly indicates how changes in musical responses are related to and can lead to cognitive enhancement. In addition, the evaluation and training protocols that we designed are readily applicable to music therapy in medical and rehabilitation settings if the use of electronic media and information technologies readily gains acceptance.

## Author contributions

EJ contributed to the conception and design of the VR-MAT. J-HS contributed to the clinical design and clinical assessments. EJ and J-HS drafted the manuscript, critically reviewed the manuscript for important intellectual content. All authors have read and approved the final manuscript.

## Funding

This work was supported by the Ministry of Education of the Republic of Korea and the National Research Foundation of Korea (NRF-2018S1A5A2A03034582) and the Technology Innovation Program (or Industrial Strategic Technology Development Program-Technology Innovation Program) (20014480, A light-weight wearable upper limb rehabilitation robot system and untact self-training and assessment platform customizable for individual patient) funded By the Ministry of Trade, Industry & Energy (MOTIE, Korea).

## Conflict of interest

The authors declare that the research was conducted in the absence of any commercial or financial relationships that could be construed as a potential conflict of interest.

## Publisher’s note

All claims expressed in this article are solely those of the authors and do not necessarily represent those of their affiliated organizations, or those of the publisher, the editors and the reviewers. Any product that may be evaluated in this article, or claim that may be made by its manufacturer, is not guaranteed or endorsed by the publisher.

## Footnotes

^1^ https://www.r-project.org/
